# Loss of FoxO3a prevents aortic aneurysm formation through maintenance of VSMC homeostasis

**DOI:** 10.1038/s41419-021-03659-y

**Published:** 2021-04-07

**Authors:** Weiling Lu, Yu Zhou, Shan Zeng, Lintao Zhong, Shiju Zhou, Haoyu Song, Rongming Ding, Gaojun Zhong, Qingrui Li, Yuhua Hu, Zhongyu Wen, Qin Liao, Yalan Wang, Lianglliang Lyu, Yiming Zhong, Gonghua Hu, Yulin Liao, Dongming Xie, Jiahe Xie

**Affiliations:** 1 Department of Cardiology, Key Laboratory of Prevention and Treatment of Cardiovascular and Cerebrovascular Diseases, Ministry of Education, First Affiliated Hospital of Gannan Medical University, Gannan Medical University, University Town, Ganzhou Development District, 341000 Ganzhou, China; 2 Department of Cardiology, Ganzhou Municipal Hospital, 49th, Grand Highway, 341000 Ganzhou, China; 3 Division of Vascular Surgery, National-Local Joint Engineering Laboratory of Vascular Disease Treatment, Engineering and Technology Center for Diagnosis and Treatment of Vascular Diseases, Guangdong Engineering Laboratory of Diagnosis and Treatment of Vascular Disease, The First Affiliated Hospital, Sun Yat-sen University, Guangzhou, China; 4 Department of Cardiology, Zhuhai People’s Hospital (Zhuhai Hospital Affiliated with Jinan University), 519000 Zhuhai, China; 5 Wards of Cadres, Zhuhai People’s Hospital (Zhuhai Hospital Affiliated with Jinan University), 519000 Zhuhai, China; 6 Department of Cardiology, State Key Laboratory of Organ Failure Research, Nanfang Hospital, Southern Medical University, 1838 North Guangzhou Avenue, 510515 Guangzhou, China; 7 Jiangxi Branch Center of National Geriatric Disease Clinical Medical Research Center, Gannan Medical University, University Town, 341000 Ganzhou Development District, Jiangxi Province China

**Keywords:** Cell biology, Diseases

## Abstract

Vascular smooth muscle cell (VSMC) phenotypic switching plays a critical role in the formation of abdominal aortic aneurysms (AAAs). FoxO3a is a key suppressor of VSMC homeostasis. We found that in human and animal AAA tissues, FoxO3a was upregulated, SM22α and α-smooth muscle actin (α-SMA) proteins were downregulated and synthetic phenotypic markers were upregulated, indicating that VSMC phenotypic switching occurred in these diseased tissues. In addition, in cultured VSMCs, significant enhancement of FoxO3a expression was found during angiotensin II (Ang II)-induced VSMC phenotypic switching. In vivo, FoxO3a overexpression in C57BL/6J mice treated with Ang II increased the formation of AAAs, whereas FoxO3a knockdown exerted an inhibitory effect on AAA formation in ApoE^−/−^ mice infused with Ang II. Mechanistically, FoxO3a overexpression significantly inhibited the expression of differentiated smooth muscle cell (SMC) markers, activated autophagy, the essential repressor of VSMC homeostasis, and promoted AAA formation. Our study revealed that FoxO3a promotes VSMC phenotypic switching to accelerate AAA formation through the P62/LC3BII autophagy signaling pathway and that therapeutic approaches that decrease FoxO3a expression may prevent AAA formation.

## Introduction

An abdominal aortic aneurysm (AAA) is defined as regional dilation of the aorta of >50% of the diameter of the normal adjacent aortic tissue or focal dilation of ≥3 cm compared to the diameter of the normal adjacent arterial segment^1–3^. The prevalence of AAAs increases with age and is 4 to 8% in men and 0.5% to 1.5% in women^4^. Patients with large aneurysms (aneurysms with a diameter of 5.0 to 5.5 cm) are at high risk for rupture and are recommended to undergo open or interventional repair^5^. AAAs smaller than 5.5 cm in diameter are termed “small AAAs”^6^. Lifestyle changes and close observation are recommended for patients with small aneurysms due to the lack of effective pharmacotherapy options^6^. Therapeutic targets for AAAs have long been sought; thus, we aimed to elucidate the pathogenesis of the disease.

Vascular smooth muscle cells (VSMCs) are remarkably plastic and can undergo dedifferentiation from the quiescent, contractile type to the proliferative, synthetic type in response to endogenous or exogenous stimuli^7^. This process is characterized by downregulated expression of contractile phenotype markers, such as SM22α and α-smooth muscle actin (α-SMA), and upregulation of synthetic phenotype markers, such as osteopontin (OPN). VSMC phenotypic switching is a characteristic feature of various vascular remodeling diseases, such as atherosclerosis^8–10^ and neointimal hyperplasia after vascular injury^11,12^. Recently, mounting evidence has suggested that VSMC phenotype transitions play a key role in the formation and progression of AAA^13–17^. Previous studies have shown that VSMC phenotype transitions are an early event of AAA^13,15,18^ and that key regulators of VSMC contractile phenotype markers, such as KLF4^9^, unspliced XBP1^13^, zinc-finger protein 148 (ZFP148)^17^, and forkhead box class O (FoxO)4^13^, are involved in AAA formation by manipulating VSMC phenotype transitions. These studies indicate that unraveling the molecular mechanisms that govern the phenotypic switching of VSMCs is of great clinical importance for AAA treatment. Despite recent advances in the recognition of VSMC phenotype transition in AAA, the regulatory mechanism has not yet been fully defined.

Compelling evidence suggests that the PI3K/Akt signaling pathway plays a pivotal role in maintaining the contractile phenotype of VSMCs in various vascular diseases^19–21^. The effect of the PI3K/Akt pathway on phenotypic modulation of VSMCs is partially dependent on the suppression of FoxO transcription factor activity^21–23^. FoxO transcription factors act as downstream targets of the PI3K/Akt pathway^21,23–25^, which induces phosphorylation of the FoxO protein and inhibition of FoxO activities. Among the FoxO transcription factors, FoxO3a and FoxO4 have been shown to repress the regulation of VSMC contractile marker genes^21,23,26–28^. Recently, a study revealed that the XBP1u-FoxO4-myocardin axis plays a pivotal role protecting against aortic aneurysm formation by maintaining the contractile phenotype of VSMCs, which indicates a novel role for FoxO transcription factors in vascular disease^13^. Interestingly, our preliminary results showed that the expression of FoxO3a is significantly increased in AAA specimens. These findings suggest that FoxO3a may be involved in the formation of AAAs. Here, we aimed to investigate the potential role of FoxO3a in angiotensin II (Ang-II)-induced AAA formation and the underlying mechanisms.

## Materials and methods

The data, analytic methods and study materials are available from the corresponding author upon reasonable request. This study was approved by the Ethical Committee of Nanfang Hospital, Southern Medical University (Guangzhou, China).

### Experimental animals

The protocol was performed following the guidelines approved by the Institutional Animal Care and Use Committee of Southern Medical University. All animal care and experimental protocols were in compliance with the National Institutes of Health guidelines for the care and use of laboratory animals. Male mice (12–13- weeks old) were used in this study. Male C57BL/6J mice with normal lipid metabolism and male apolipoprotein E-deficient (ApoE^−/−^) mice on the C57BL/6J background were provided by the Experimental Animal Center of Southern Medical University. All mice used in the study were fed normal mouse food. The mice were kept under pathogen-free conditions and maintained at a standard temperature and humidity.

### Transfection

AAV-FoxO3a, sh-FoxO3a and negative control (scramble) were synthesized and purchased from Vigene. AAV-FoxO3a, AAV-sh-FoxO3a or AAV-scramble (1 × 10^11^ vector genomes) was injected into the mice through the tail vein, and after 4 weeks, the mice were infused with AngII or physiological saline for 28 days. The full details of the AAV constructs is available in the Supplemental Figure (Supplemental Fig. 4)

### Ang II-induced AAA model

Eight- to 15-week-old male wild-type mice, 10- to 16-week-old male ApoE^−/−^ mice and 8- to 15-week-old male C57BL/6J mice were used in these studies. An osmotic minipump (Alzet, Model 2004; DURECT Corporation, Cupertino, CA) was subcutaneously implanted in the dorsum of the neck via a small incision; Ang II (A9525; Sigma, St. Louis, MO) or normal saline was infused via the minipump at the well-established rate of 1 μg/kg per minute for 28 days. Mice were euthanized via an overdose of sodium pentobarbital (150 mg/kg, intraperitoneal injection), and their aortas were collected. Animals that died of aortic rupture were used to calculate the mortality and rupture rate only and were excluded from the tissue degradation analysis.

### Aneurysm quantification

The suprarenal abdominal aorta referred to the part between the last pair of intercostal arteries and the right renal branch. The outer diameter of the maximal dilated portion of the suprarenal aorta was measured as the maximal aortic diameter by a coworker blinded to the group assignment using Image-Pro Plus (IPP) software (Media Cybernetics) according to the aorta digital images. Aneurysm formation was identified as an increase in the outer width of the suprarenal aorta by at least 50% or greater compared with the adjacent nonaneurysmal section.

### Histology and immunohistochemistry

Murine and human aortas were fixed in 4% formalin and embedded in paraffin according to standard protocols. Hematoxylin and eosin (H&E) staining was performed for morphological assessment. For immunohistochemistry, a rabbit anti-FoxO3a antibody (1:200; Cell Science, USA), donkey anti-α-SMA antibody (1:200; Abcam, UK), rabbit anti-SM-22α antibody (1:200; Abcam, UK), and rabbit anti-Osteopontin antibody (1:200; Abcam, UK) were used. Paraffin sections of murine and human aortas were dewaxed and hydrated, and endogenous peroxidase activity was blocked with 3% hydrogen peroxide for 30 min. The sections were incubated with 5% bovine serum for 1 h at room temperature to block nonspecific binding sites followed by primary antibody at 4 °C overnight and secondary antibody (1:500; Cell Science, USA) for 60 min. All specimens were stained with DAB and hematoxylin staining solution. A minimum of three microscopic fields of stained slides were randomly observed by two independent researchers who were unaware of the group information.

### Immunohistochemical staining protein quantification

Protein quantification was determined using IPP software (MEDIA CYBERNETICS, USA), based on the integral optical density (IOD) value, the sum of the chroma and the acreage of positive color (claybank). The mean IOD was calculated as the IOD value divided by the acreage of the samples. Each specimen had 3 lower power sections and 9 higher power sections. The mean IOD was the mean value of the IOD value in the 9 higher power sections.

### Immunofluorescence microscopy (F-actin)

Aortic VSMCs between passage 3 and 6 that had been cultured in serum-free medium for 24 h and treated with AngII for 24 h were used in this study. VSMCs were seeded on confocal dishes and fixed in 4% paraformaldehyde for 30 min at room temperature. The cells were then rinsed 3 times in PBS and incubated with PBS containing 0.1% Triton X-100 (PBS) for 2 min at room temperature. The cells were then rinsed with PBS and incubated with Actin-Tracker Green (Beyotime, China) for 1 h at room temperature. The cells were then incubated with DAPI for 15 min. Finally, the cells were washed with PBS, and images were acquired using a confocal microscope (Leica, Germany).

### Cell culture and treatment

Aortic VSMCs (MOVAS-1) were purchased from Guangzhou Geneseed Biotech, Ltd. The VSMCs were kept in DMEM containing 4.5 g glucose, fetal bovine serum (10%), 100 U/mL penicillin, and 100 μg/mL streptomycin and cultured in a humidified environment containing 5% CO_2_ at 37 °C. Cells between passage 3 and passage 6 that had been cultured in serum-free medium for 24 h and had reached 70 to 80% confluence were treated with AngII (1 μmol/L).

The small-interfering RNA (siRNA) sequences against FoxO3a and the overexpression plasmids FoxO3a were synthesized by Vigene Bioscience (Jinan, Shandong, China). VSMC were seeded into 6-well plates at 50 to 70% confluence, cells were starved with DMEM without FBS or penicillin/streptomycin for 24 h. Cells were transfected with si-FoxO3a (50 nM) or overexpression plasmid FoxO3a (3 μg). After a 12-h incubation, cells were cultured in DMEM supplemented with FBS and penicillin/streptomycin. After 24 h of growth, the cells were treated with AngII (1 μmol/L).

### Measurement of smooth muscle cell (SMC) migration by the wound scratch assay

A total of 5 × 10^4^ VSMCs were seeded in 6-well plates, and when the percentage of cell fusion reached ≈80%, the VSMCs were cultured in serum-free medium for 24 h. Scratch wounds were made by scraping the cell layer on each plate using sterile micropipette 200 μl pipette tips. The isolated cells were washed with PBS and removed, and then the VSMCs were stimulated with AngII (1 μmol/L), a proliferation inhibitor (10 mol Mitomycin C, GlpBio, USA) or PBS control for 8 h in medium containing 5% serum. Images were taken under a microscope after 0 and 8 h and analyzed using ImageJ. Using recovered areas/scratched areas.

### Elastin staining and degradation

Kidney and aortic samples from different groups of mice were embedded in paraffin, dewaxed, hydrated, and then subjected to Victoria Blue Van Giessen staining using a commercial kit (GenMed, Shanghai). Elastin degradation was assessed at 40x magnification. Severity was assessed according to the following previously established elastin degradation scoring criteria: (1) inelastic protein degradation and a good elastin layer; (2) mild degradation of elastin and some laminar disruption or rupture; (3) moderate elastin degradation and multiple interruptions or breaks in the lamina; (4) severe elastin breakdown or loss or aortic rupture.

### Western blotting

Human and mouse aortic tissues and VSMCs were lysed with RIPA lysis buffer (Fude Biological, China), and then the protein concentration was measured using a BCA protein assay kit (Beibo, China). High-temperature denaturation and protein separation were performed by sodium dodecyl sulfate-polyacrylamide gel electrophoresis. The proteins were then transferred to polyvinylidene fluoride (PVDF) membranes with 0.22-μm pores (Millipore, MA, USA). The membranes were washed in 0.1% Tween diluted in Tris-buffered saline, blocked with 5% BSA for 1 h and then incubated with primary antibody at 4 °C overnight. All primary antibodies were diluted with primary antibody dilution buffer. Rabbit anti-FoxO3a (1:1000; Cell Science, USA), donkey anti-α-SMA (1:1000; Abcam, UK), rabbit anti-SM-22α (1:1000; Abcam, UK), rabbit anti-MMP-2 (1:1000; Abcam, UK), rabbit anti-P62 (1:1000; Cell Science, USA), rabbit anti-LC3β (1:1000; Cell Science, USA) and rabbit anti-FoxO1a (1:1000; Abcam, UK) primary antibodies were used. Next, the membranes were washed and incubated with horseradish peroxidase-conjugated secondary antibody (donkey ant-goat antibody, 1:5000; Arigo, China; or anti-rabbit IgG antibody, 1:5000; Cell Science, USA) for 1 h. Finally, the membranes were washed and treated with FDbio-Dura ECL chemiluminescence solution. Protein expression analysis was then performed using ImageJ Analysis software (National Institutes of Health, Bethesda, MD).

### Statistical analysis

All data are presented as the mean and standard error or median and interquartile range (the latter was used for the elastin degradation score). The data were analyzed using GraphPad Prism 7.0 and SPSS 14.0. Normality tests were assessed by Shapiro–Wilk statistics. When the data were normally distributed, Student’s *t*-tests were applied to determine statistical significance between two groups. Differences among groups were determined by one-way ANOVA, followed by Least significant difference post hoc test or Dunnett’s test. If a normal distribution could not be confirmed, nonparametric tests were applied using the Mann-Whitney *U*-test for two independent groups or Kruskal–Wallis test with post hoc Dunn’s multiple comparisons test for ≥3 groups. Fisher exact test was used to analyze the aneurysm incidence. *p* < 0.05 was considered statistically significant.

## Results

### FoxO3a expression is upregulated in human AAA tissues

**Fig. 1:**
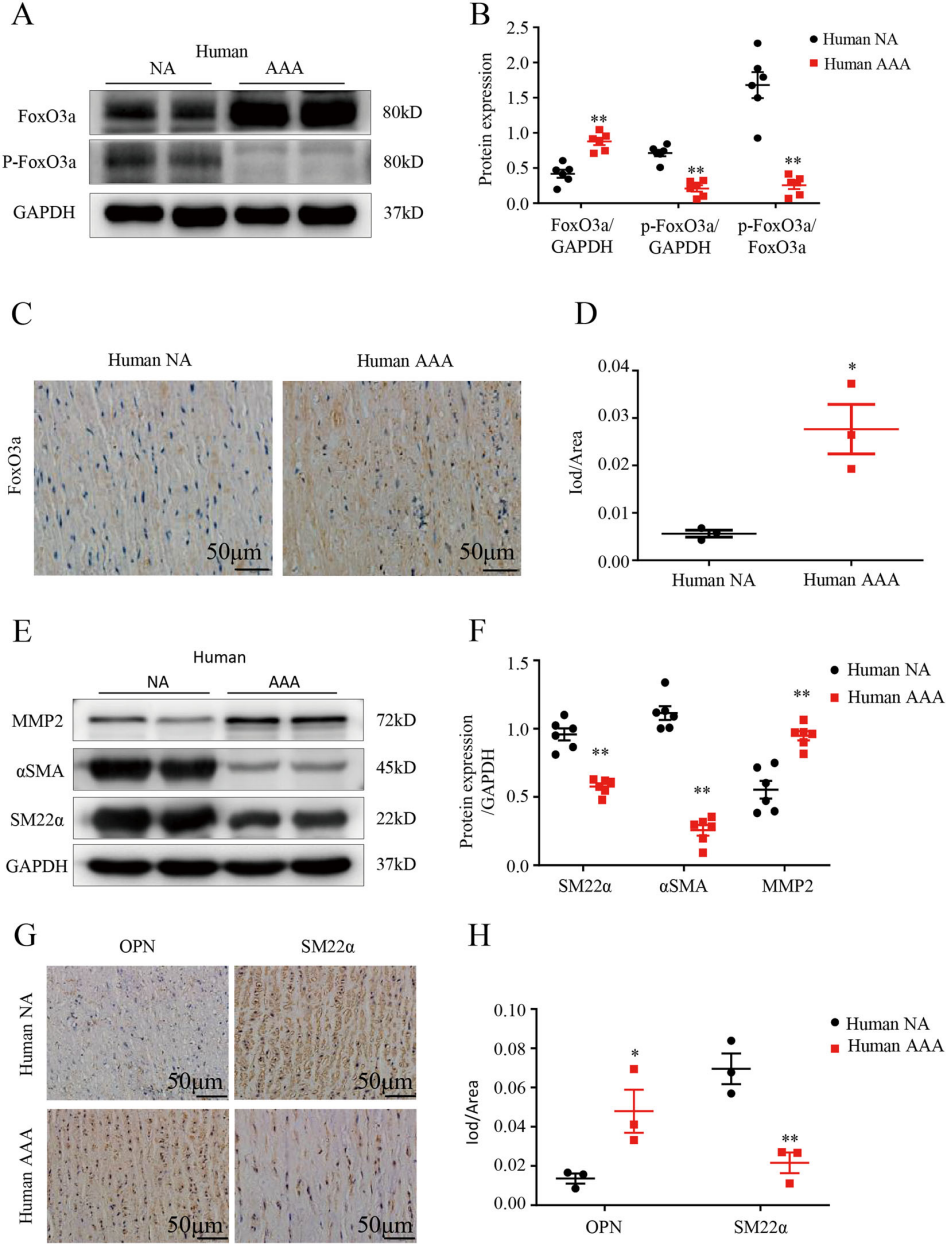
FoxO3a is upregulated in human tissues and the VSMC phenotypic switch occurs during abdominal aortic aneurysm (AAA) formation. **A** and **B** Western blots (WB) and densitometric analysis of the protein levels of Forkhead box class O3a (FoxO3a) and phospho Forkhead box class O3a (p-FoxO3a) in human AAA samples and adjacent nonaneurysmal aortic sections (male; *n* = 6). **C** and **D** Representative FoxO3a staining in human AAA samples and adjacent control aortas (scale bars = 50 µm, male; *n* = 3). **E** and **F** WB and densitometric analysis of the protein levels of α-smooth muscle actin (α-SMA), matrix metalloproteinase (MMP2) and smooth muscle 22α (SM22α) in human AAA samples and adjacent nonaneurysmal aortic sections (male; *n* = 6). **G** and **H** Representative staining with osteopontin (OPN) and SM22α in human AAA samples and adjacent control aortas (scale bars = 50 µm, male; *n* = 3). Data are presented as the mean ± SE. *p < 0.05, **p < 0.01. NA indicates nonaneurysmal aorta.

To elucidate the potential role of FoxO3a in the development of aneurysms, AAA tissues from patients undergoing AAA resection and corresponding adjacent normal aortic tissues were examined. Western blotting showed that the protein expression of FoxO3a in AAA tissues was significantly higher than that in the corresponding normal aortic tissues and that the protein expression of p-FoxO3a was significantly decreased (*p* < 0.01; Fig. 1A, B). Immunohistochemical staining confirmed that the level of FoxO3a in human active SMCs was significantly higher than that in adjacent nonaneurysmal SMCs (*p* < 0.05; Fig. 1C, D). As expected, we found that the protein levels of the contractile SMC markers SM-22α and α-SMA were significantly downregulated in AAA tissues compared to normal tissues, while extracellular matrix proteolytic enzyme 2 (MMP2), a synthetic phenotype marker, was significantly upregulated (*p* < 0.01; Fig. 1E, F). Immunohistochemical analysis revealed that the expression of the synthetic phenotype marker OPN was significantly higher (*p* < 0.05) and that SM22α expression was significantly lower (*p* < 0.01) in human AAA tissue than in adjacent nonaneurysmal tissue (Fig. 1G, H). These results are consistent with the finding that upregulation of FoxO3a expression is accompanied by phenotypic changes in VSMCs in human AAA.

### FoxO3a expression is upregulated in tissues from a mouse model of Ang II-Induced AAA

**Fig. 2:**
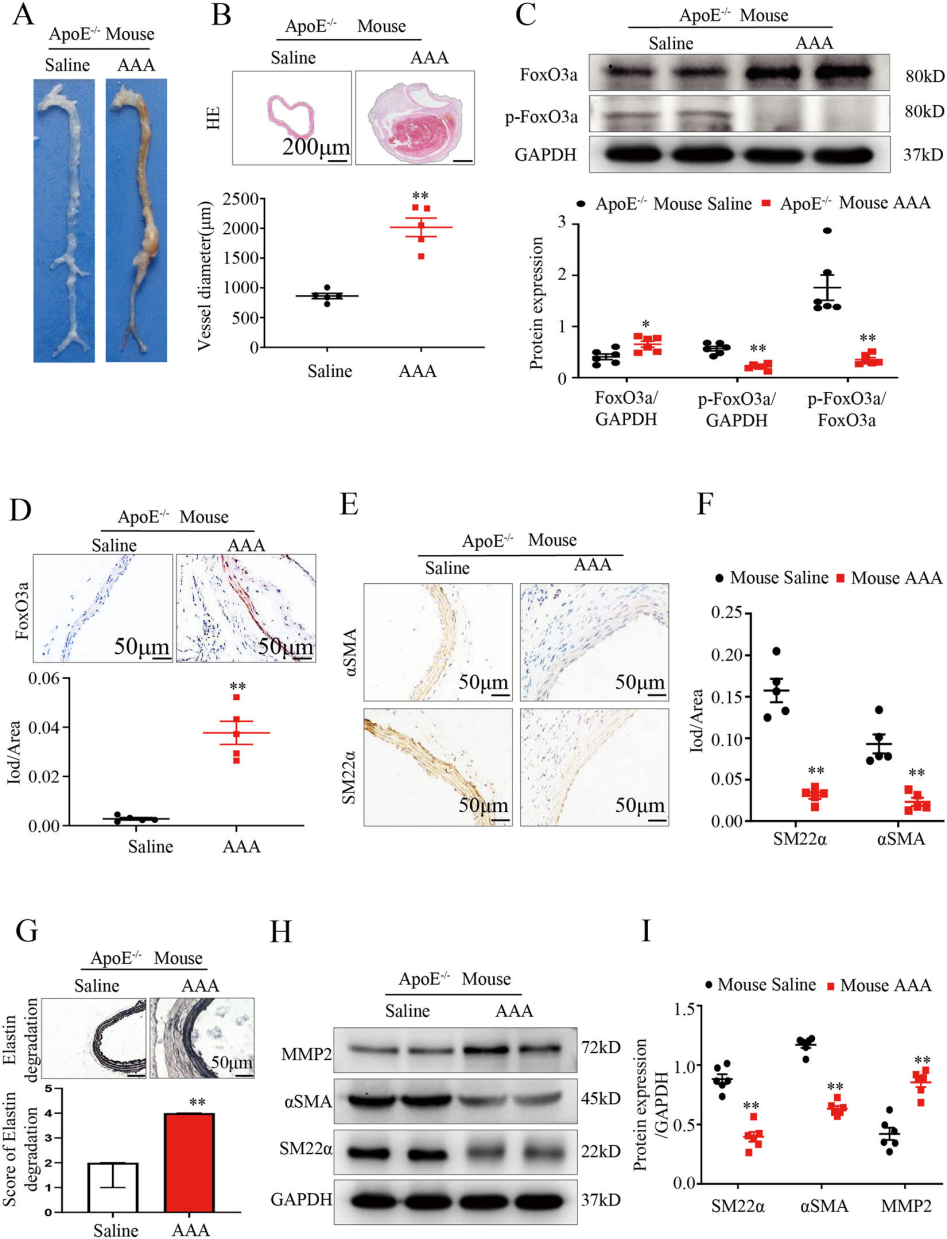
FoxO3a is upregulated in the aneurysm tissues of apolipoprotein E–deficient (ApoE^−/−^) mice induced by angiotensin II (Ang II) and the VSMC phenotypic switch occurs during AAA formation. **A** Representative photographs showing the macroscopic features of aortic aneurysms at 28 d after Ang II infusion in male ApoE^−/−^mice. **B** Representative pictures of aorta cross-sections stained with hematoxylin-eosin (scale bar = 200 μm) and the vessel diameter (μm) in aortic aneurysms (AAs) from Ang II-induced male ApoE^−/−^ mice and control male ApoE^−/−^ mice (*n* = 5). **C** WB and densitometric analysis of the protein levels of FoxO3a and p-FoxO3a in AAs from Ang II-induced male ApoE^−/−^ mice and control male ApoE^−/−^ mice (male; *n* = 6). **D** FoxO3a immunohistochemical staining of the aortas in AAs from Ang II-induced male ApoE^−/−^ mice and control male ApoE^−/−^ mice (scale bar = 50 μm; *n* = 5). **E** and **F** Representative immunohistochemical staining of α-SMA and SM22α in suprarenal AAs from male ApoE^−/−^ mice treated with Ang II and control male ApoE^−/−^ mice (scale bar = 50 μm; *n* = 5). **G** Representative staining of elastin in the abdominal aortas of angiotensin-infused male ApoE^−/−^ mice (*n* = 15). **H** and **I** WB and densitometric analysis of the protein levels of α-SMA, MMP2 and SM22α in the AAs of Ang II-induced male ApoE^−/−^ mice and control male ApoE^−/−^ mice (*n* = 6). Data are presented as the mean ± SE. *p < 0.05, **p < 0.01. Saline indicates control male ApoE^−/−^ mice.

We further investigated the expression of FoxO3a in a mouse model of AAA constructed by infusing ApoE^−/−^ mice with Ang II. Aneurysm formation 28 days after infusion in these mice (Fig. 2A) but not in control mice (Supplemental Fig. 2A) indicated successful AAA modeling. The blood vessel diameter of model mice that developed aneurysms was 50% higher than of the control group, which is similar to the change in diameter observed for human aneurysms (*p* < 0.01; Fig. 2B). Western blotting showed that the protein expression of FoxO3a was significantly higher and that the protein expression of p-FoxO3a was significantly lower in tissues from mice with Ang II-induced AAA than in control mice (*p* < 0.01; Fig. 2C). Immunohistochemical staining confirmed that the level of FoxO3a was significantly higher in the AAA model mice than the control mice (Fig. 2D).

Histological analysis showed that the SMC phenotypic process in mouse AAA tissues was consistent with that in human AAA. Compared with those in the control group, the levels of SM22α and α-SMA in SMCs from mouse AAA tissues were significantly lower (*p* < 0.01; Fig. 2E, F). Correspondingly, the degree of elastin degradation in mouse AAA tissues was more severe than in the control group (Fig. 2G). Western blotting showed that compared with that in the control group, the expression of the contractile proteins SM-22α and α-SMA in the model group was significantly downregulated, while the extracellular matrix proteolytic enzyme MMP2, which promotes VSMC migration, was upregulated (*p* < 0.01; Fig. 2H, I) This findings suggest that mouse AAA is similar to human AAA and are consistent with the findings related to SMC phenotypic transition.

### FoxO3a expression is upregulated during phenotypic switch induced by Ang II in vitro

**Fig. 3:**
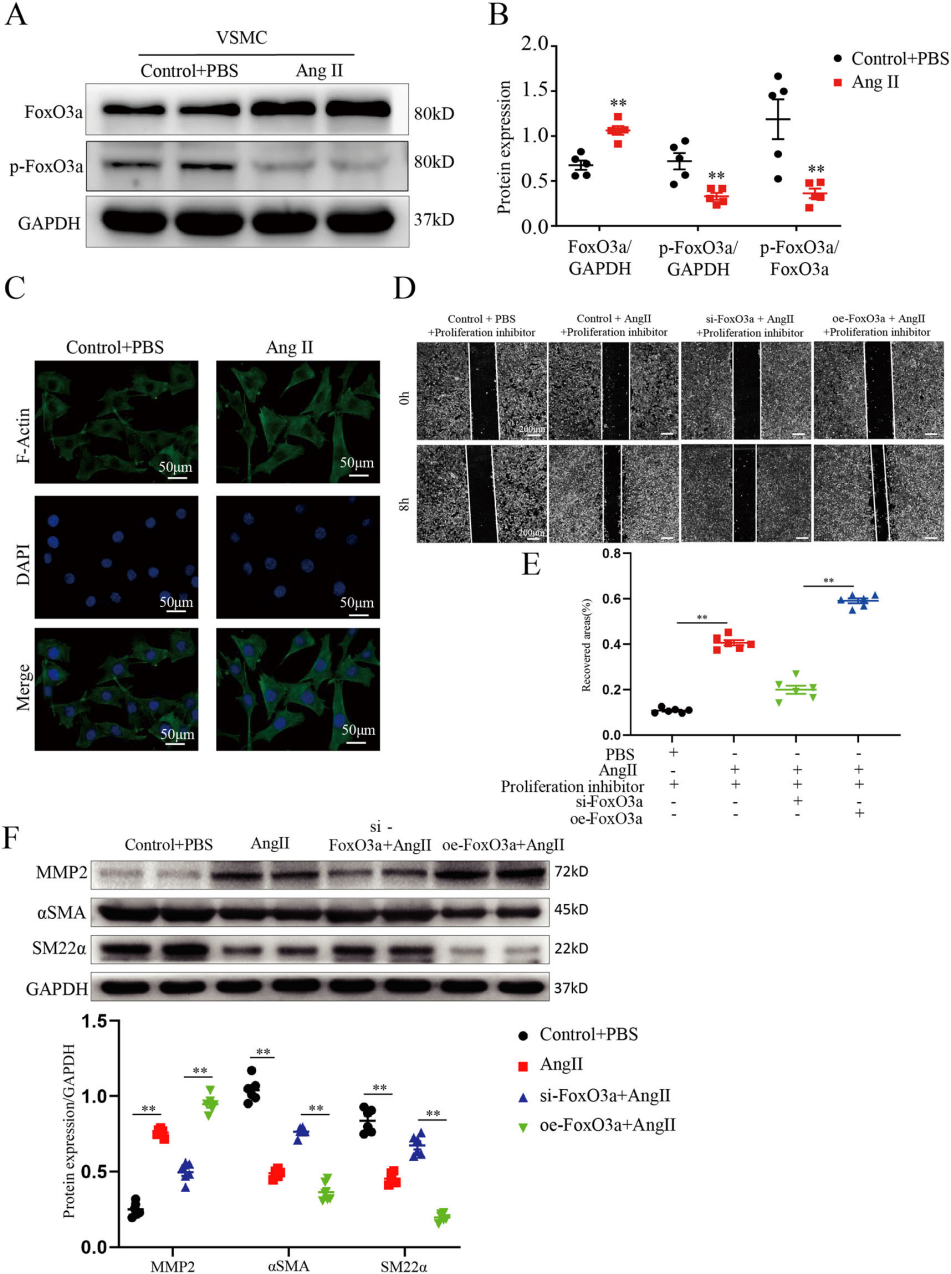
FoxO3a expression is upregulated during the phenotypic switch induced by Ang II in vitro. **A** and **B** WB and densitometric analysis of the protein levels of FoxO3a and p-FoxO3a in VSMCs treated with Ang II for 24 h (1 μmol/L; *n* = 6). **C** Representative pictures of VSMCs stained with actin-tracker green (treated with Ang II for 24 h; 1 μmol/L; DAPI, blue; actin-tracker green, green). **D** and **E** Wound scratch assay was performed to assess VSMC motility and migration within VSMCs after Ang II administration and/or functional FoxO3a knockdown or overexpression (treated with Ang II for 0 h or 8 h; 1 μmol/L). **F** WB and densitometric analysis of the protein levels of α-SMA, MMP2 and SM22α within VSMCs after Ang II administration and/or functional FoxO3a knockdown or overexpression (*n* = 6). Data are presented as the mean ± SE. *p < 0.05, **p < 0.01. Control indicates VSMCs treated with PBS. oe, overexpression; si,siRNA.

VSMCs treated with AngII are classic cell models of AAA. Previous studies have found that Ang II can induce phenotypic switching of VSMCs. To study the role of FoxO3a in VSMC phenotype switching, VSMCs were treated with Ang II for 24 h. VSMCs that transform from a contracted phenotype to a synthetic have high proliferation and migration abilities, which contribute to the progression of AAA. MMPs mediate extracellular matrix degradation and promote VSMC migration^29^. We found that FoxO3a protein expression was significantly higher and p-FoxO3a protein expression was lower in VSMCs treated with Ang II than in control VSMCs (*p* < 0.01; Fig. 3A, B). Contractile markers of differentiated SMCs (SM22α and α-SMA) were synergistically downregulated, while MMP2 was upregulated in Ang II-treated VSMCs compared to control VSMCs (*p* < 0.01; Supplemental Fig. 3A, B). The formation and enrichment of F-actin stress fibers contributes to VSMC migration and proliferation^30,31^. Ang II induced VSMC F-actin stress fiber formation (Fig. 3C) and significant VSMC migration at 8 h (Supplemental Fig. 3C). Correspondingly, our results showed that knockdown of FoxO3a significantly inhibited MMP2, but increased SM-22α and α-SMA expression, while overexpression of FoxO3a showed the opposite effect (Fig. 3D–F). These results suggest that FoxO3a enhances VSMC migration, possibly by regulating VSMC phenotypic switching and MMP-2 expression.

### Overexpression of FoxO3a promotes AAA formation in Ang II-Infused C57BL/6J mice

**Fig. 4:**
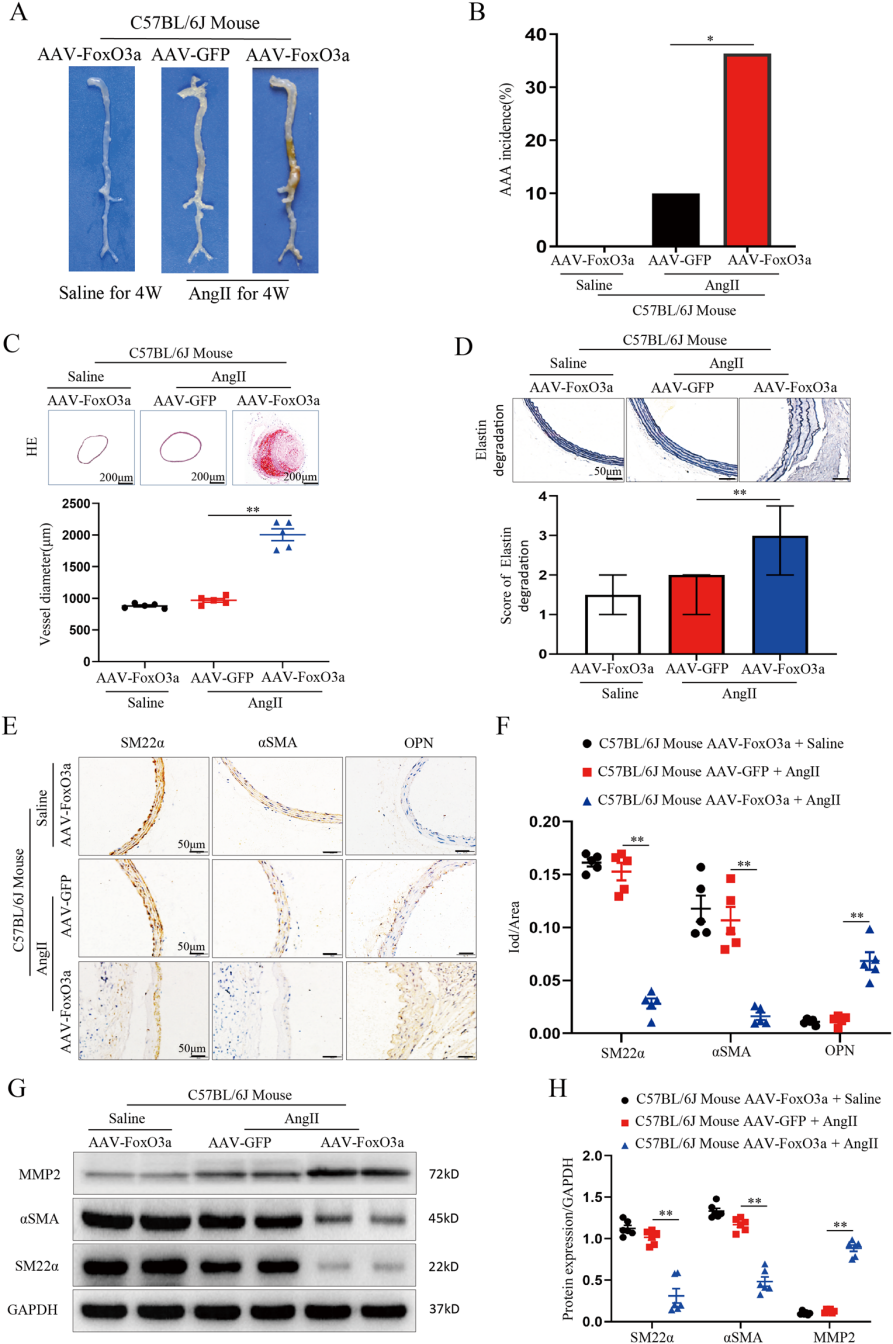
FoxO3a overexpression promotes AAA formation in Ang II-infused C57BL/6J mice. Male C57BL/6J mice were treated with saline or AAV- FoxO3a or AAV-GFP, followed by infusion with saline or Ang II for 28 days. Data are presented as the mean ± SE; *n* = 10 in the AAV- FoxO3a group (mice treated with saline), *n* = 30 in the AAV-FoxO3a or AAV-GFP group (mice treated with Ang II). **A** Representative photographs showing AAAs in 3 groups of Ang II-treated C57BL/6J mice. **B** Statistical analysis of the AAA incidence. **C** Hematoxylin-eosin staining and maximal diameter of the abdominal region in C57BL/6J mice (*n* = 5). **D** Representative staining with elastin in the abdominal aortas in 3 groups of C57BL/6J mice. Photographs show the location where the most severe elastin degradation occurred (scale bars = 50 µm; magnified photographs, *n* = 15). **E** and **F** Representative staining of the SM22α, α-SMA and OPN proteins (scale bars, 50 µm, *n* = 5). **G** and **H** WBs and densitometric analysis of aortic MMP2, α-SMA and SM22α in C57BL/6J mice (*n* = 6). Data are presented as the mean ± SE. *p < 0.05, **p < 0.01.

To further identify a potential causative link between FoxO3a overexpression and AAA development, we used an AAV carrying a FoxO3a overexpression plasmid to perform gain-of-function studies in Ang II-perfused C57BL/6J mice treated with AAV-FoxO3a. 30 days after injection of AAV virus, the overexpression interventions yielded significant and continuous promotion of FoxO3a expression, compared with saline group (Supplemental Fig. 1). Therefore, on the 30th day after AAV administration, C57BL/6J mice were randomly selected to receive saline or Ang II infusion for 28 days. As expected, FoxO3a-overexpressing C57BL/6J mice treated with saline showed no AAA formation. After 28 days of Ang II infusion, there was a substantial increase in AAA formation in the AAV-FoxO3a group (11 of 30 mice (36.6%) in the AAV-FoxO3a group developed AAAs; *p* < 0.05; Fig. 4A, B) compared with the AAV-GFP group, in which AAA formation was nearly absent (3 of 30 mice (10%) in the AAV-GFP group developed AAAs; *p* < 0.05; Fig. 4A, B). The blood vessel diameter of mice in the AAV-FoxO3a group that developed aneurysms was 50% higher than that of mice in the AAV-GFP group (*p* < 0.01; Fig. 4C). Correspondingly, AngII-induced elastin degradation in the AAV-FoxO3a group was more severe than that in the sham AAV-GFP group (*p* < 0.01; Fig. 4D). The expression of the contractile markers α-SMA and SM22α was lower, and the expression of the synthetic marker OPN was higher in the AAV-FoxO3a group than in the AAV-GFP group (*p* < 0.01; Fig. 4E, F). In addition, the western blotting results showed that the aortic proteins α-SMA and SM-22α were remarkably downregulated in the AAV-FoxO3a group compared to the AAV-GFP group, and the MMP2 protein was significantly higher in the AAV-GFP group than in the AAV-FoxO3a group (*p* < 0.01; Fig. 4G, H). These results indicate that FoxO3a plays an important role in AngII-induced AAA.

### Disruption of FoxO3a inhibits AAA formation in Ang II-Infused ApoE^−/−^ mice

**Fig. 5:**
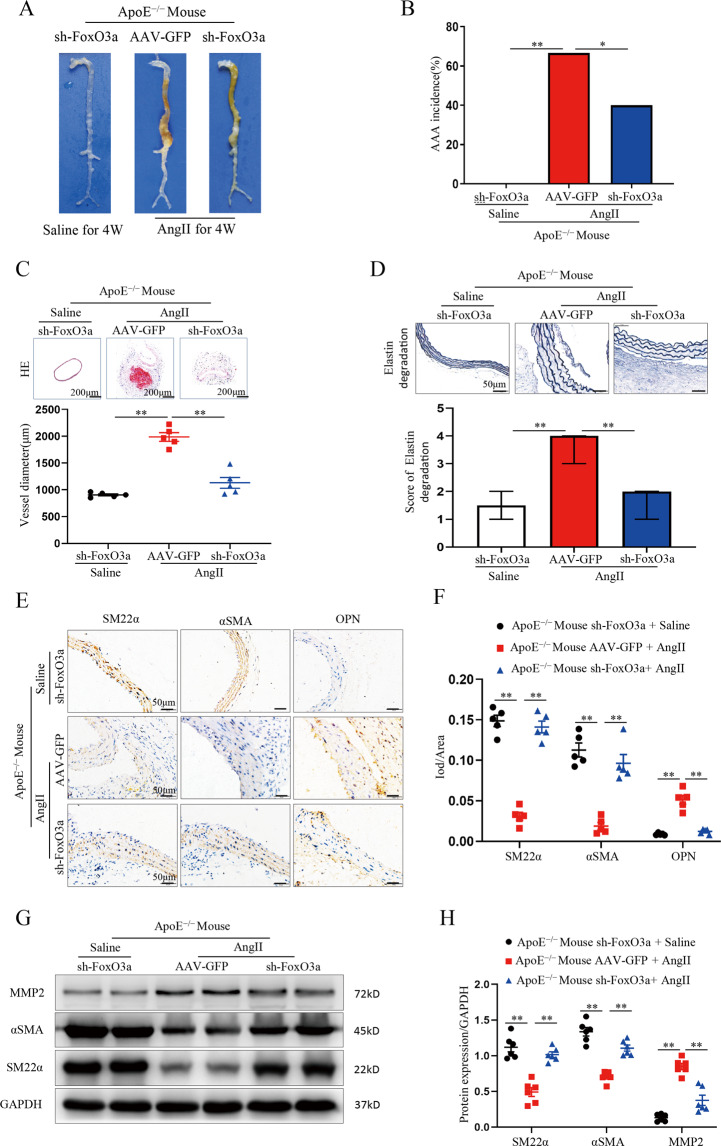
FoxO3a knockdown decreased AAA formation in Ang II-infused ApoE^−/−^ mice. Male ApoE^−/−^ mice were treated with AAV-GFP or sh-FoxO3a, followed by infusion with saline or Ang II for 28 days. Data are presented as the mean ± SE; *n* = 10 in the sh-FoxO3a group (mice treated with saline), *n* = 30 in the AAV-GFP or sh-FoxO3a groups (mice treated with Ang II). **A** Representative photographs showing AAAs in all three groups. **B** Statistical analysis of the AAA incidence in ApoE^−/−^ mice. **C** Hematoxylin-eosin staining and maximal diameter in the abdominal region (*n* = 5). **D** Representative staining with elastin in the abdominal aortas in the three groups of ApoE^−/−^ mice. Photographs show the location where the most severe elastin degradation occurred (scale bars, 50 µm; magnified photographs *n* = 15). **E** and **F** Representative staining of the SM22α, α-SMA and OPN proteins (scale bars, 50 µm, *n* = 5). **G** and **H** WBs and densitometric analysis of aortic MMP2, α-SMA and SM22α (*n* = 6). Data are presented as the mean ± SE. *p < 0.05, **p < 0.01.

Ang II induces a high rate of AAA formation in ApoE^−/−^ mice. To explore the effects of FoxO3a disruption on AAA formation, we examined the role of FoxO3a deficiency in the Ang-induced AAA model. We selected an AAV carrying a FoxO3a siRNA to perform loss-of-function studies in Ang II-perfused ApoE^−/−^ mice administered AAV-GFP or sh-FoxO3a. On the 30th day, ApoE^−/−^ mice were randomly selected to receive Ang II infusion for 28 days. ApoE^−/−^ FoxO3a- knockdown mice infused with saline were used as the control group (sh-FoxO3a treated with saline). After 28 days, the FoxO3a- knockdown mice treated with saline showed no AAA formation. AAA formation was decreased in AAV-sh-FoxO3a-treated mice compared with AAV-GFP-treated mice (12 of 30 mice (40%) in the sh-FoxO3a group developed AAAs; 20 of 30 mice (66.7%) in the AAV-GFP group developed AAAs; *p* < 0.05; Fig. 5 A, B). The blood vessel diameter of mice in the AAV-GFP group that developed aneurysms was 50% higher than that of sh-FoxO3a-treated mice (*p* < 0.01; Fig. 5C). Correspondingly, there was less elastin degradation in the sh-FoxO3 group than in the sham AAV-GFP group (*p* < 0.01; Fig. 5D). Immunohistochemical staining showed that the levels of α-SMA and SM22α were significantly upregulated and that the level of OPN was significantly downregulated in the sh-FoxO3a group compared to the AAV-GFP group (*p* < 0.01; Fig. 5E, F). Upregulation of a-SMA and SM-22a expression and downregulation of MMP-2 expression in the sh-FoxO3a group compared to the AAV-GFP group were detected by Western blotting (*p* < 0.01; Fig. 5G, H). These results indicate that knockdown of FoxO3a inhibits AAA formation and related VSMC phenotypic changes.

### Autophagy is activated during AAA formation

**Fig. 6:**
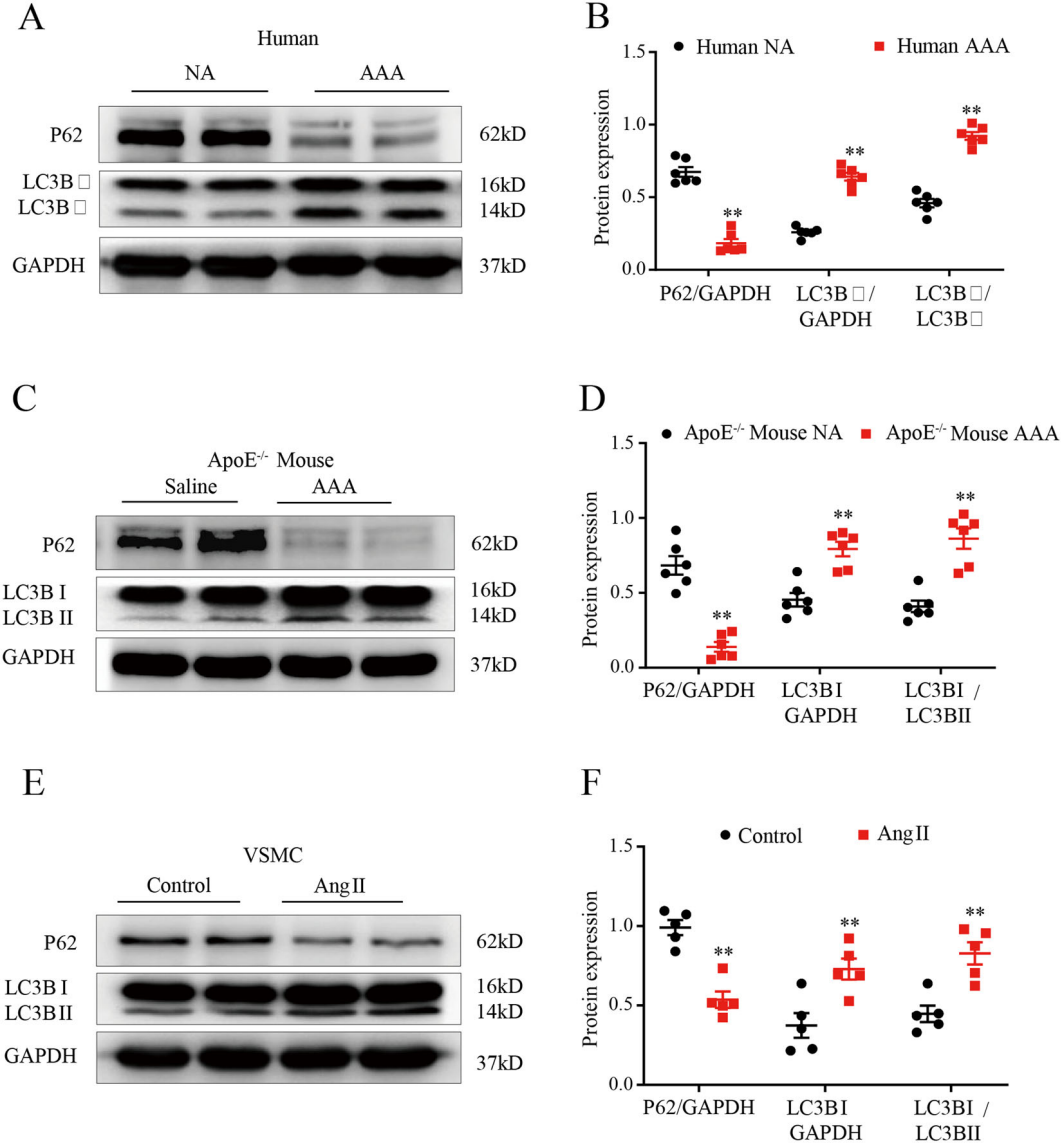
Autophagy is activated during AAA formation. **A** and **B** WBs and densitometric analysis of the protein levels of P62 and LC3β in human AAA samples and adjacent nonaneurysmal aortic sections (*n* = 6). NA indicates nonaneurysmal aorta. **C** and **D** WBs and densitometric analysis of the protein levels of P62 and LC3β in AAs from Ang II-induced ApoE^−/−^ mice and control ApoE^−/−^ mice (male; *n* = 6). Saline indicates control male ApoE^−/−^ mice. **E** and **F** WBs and densitometric analysis of the protein levels of P62 and LC3β in VSMCs treated with Ang II for 24 h (1 μmol/L; *n* = 6). Control indicates VSMCs treated with PBS. Data are presented as the mean ± SE. *p < 0.05, **p < 0.01.

Recent studies have found that autophagy is involved in AAA formation. Consistent with previous studies, we detected the expression of autophagy-related proteins in human AAA tissues and corresponding adjacent normal aortic tissues. Western blotting showed that the protein expression of P62 was significantly downregulated and that the protein expression of LC3BII was significantly upregulated in AAA tissues compared to corresponding normal aortic tissues (*p* < 0.01; Fig. 6A, B), indicating that the level of autophagy in human aneurysm tissue is significantly higher than that in adjacent tissue. We also found that the autophagy level of aneurysm tissue in the model group was higher than that in the sham group (*p* < 0.01; Fig. 6C, D), and we found the same results in VSMCs treated with Ang II in vitro (*p* < 0.01; Fig. 6E, F).

### FoxO3a promotes AAA formation by activating autophagy

**Fig. 7:**
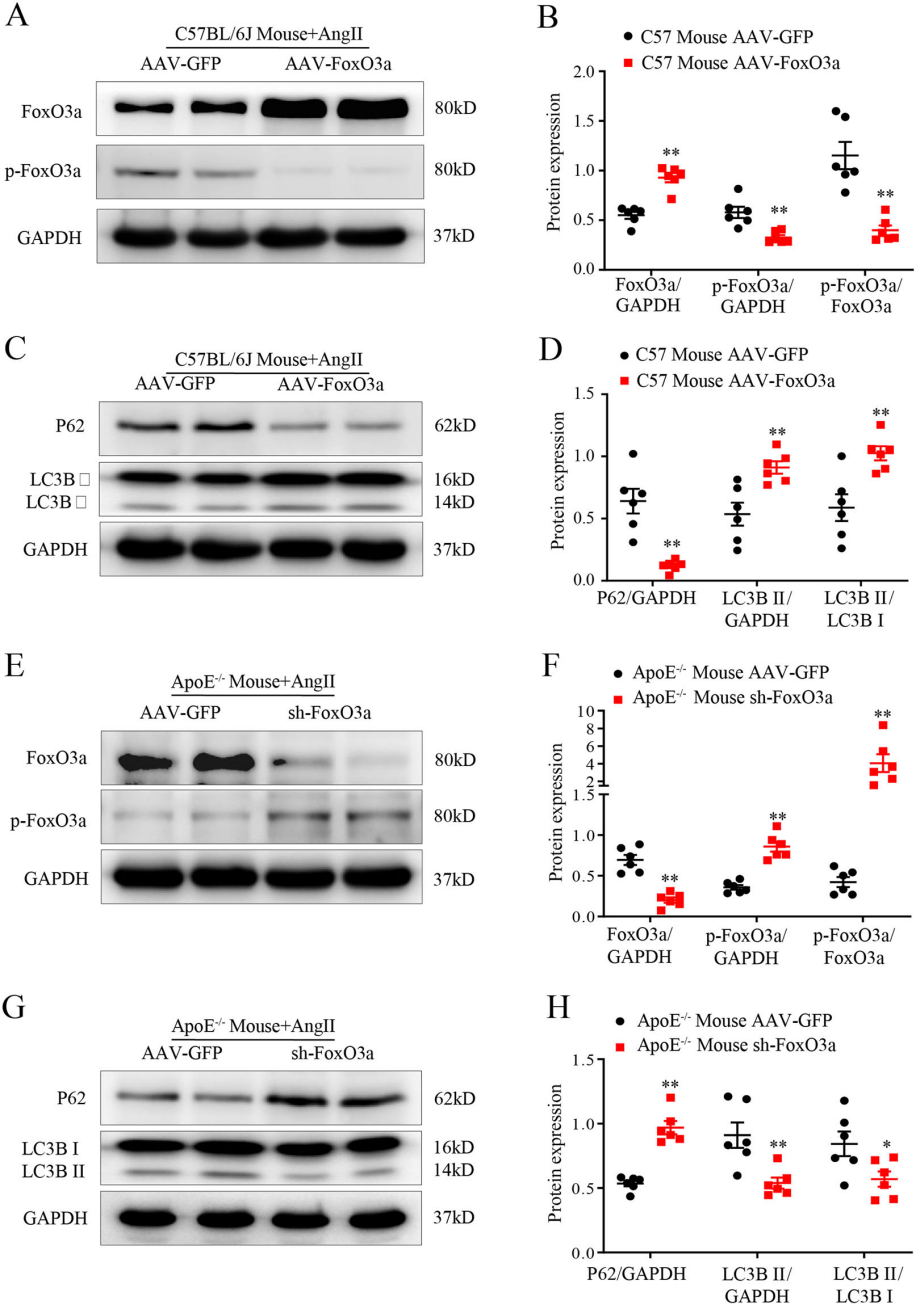
FoxO3a promotes AAA formation by activating autophagy. **A** and **B** WBs and densitometric analysis of the protein levels of FoxO3a and p-FoxO3a in AAs from Ang II-infused C57BL/6J mice. Male C57BL/6J mice were treated with Ang II for 28 days, followed by transfection with AAV-FoxO3a or AAV-GFP (*n* = 6). **C** and **D** WBs and densitometric analysis of the protein levels of P62 and LC3β in AAs from Ang II-infused C57BL/6J mice. Male C57BL/6J mice were treated with Ang II for 28 days, followed by transfection with AAV- FoxO3a or AAV-GFP (*n* = 6). **E** and **F** WBs and densitometric analysis of the protein levels of FoxO3a and p-FoxO3a in AAs from Ang II-infused ApoE^−/−^ mice. Male ApoE^−/−^ mice were treated with Ang II for 28 days, followed by transfection with AAV-GFP or sh-FoxO3a (*n* = 6). **G** and **H** WBs and densitometric analysis of the protein levels of P62 and LC3β in AAs from Ang II-infused ApoE^−/−^ mice. Male ApoE^−/−^ mice were treated with Ang II for 28 days, followed by transfection with AAV-GFP or sh-FoxO3a (*n* = 6). Data are presented as the mean ± SE. *p < 0.05, **p < 0.01.

To determine the role of the autophagy pathway in FoxO3a overexpression-mediated AAA formation, we sought to determine whether FoxO3a promotes AngII-induced VSMC phenotypic switching by activating autophagy. We overexpressed FoxO3a in C57BL/6J mice and knocked down FoxO3a in ApoE^−/−^ mice to determine the effect on autophagy levels. After 28 days of Ang II infusion, in the AAV-FoxO3a group, the protein expression level of FoxO3a was significantly higher, whereas the protein expression level of p-FoxO3a was significantly lower than that in the AAV-GFP group (*p* < 0.01; Fig. 7A. B), and autophagy was activated (*p* < 0.01; Fig. 7C, D). In contrast, in the sh-FoxO3a group, the protein expression level of FoxO3a was significantly lower, whereas the protein expression level of p-FoxO3a was significantly higher than that in the AAV-GFP group (*p* < 0.01; Fig. 7E, F), and autophagy was suppressed. (*p* < 0.01; Fig. 7G, H). A previous study suggested that an additional mechanism by which FoxO3a inhibits VSMC marker gene expression is through repressing Myocd expression in VSMCs. We also found that FoxO3a overexpression significantly inhibited Myocd expression (*p* < 0.01; Supplemental Fig. 5). These results indicate that FoxO3 promotes AngII-induced VSMC phenotype switching and AAA formation by activating autophagy and restraining Myocd expression.

## Discussion

In the current study, we demonstrated that FoxO3a acts as a novel molecular link between VSMC phenotypic switching and the initiation and development of AAA. The following major findings support this conclusion. First, the expression of FoxO3a was significantly higher in human and mouse AAA samples than in control samples. Second, knockdown of FoxO3a exerted an inhibitory effect on AAA formation in an Ang II-induced AAA model, while FoxO3a overexpression predisposed the aorta to aortic aneurysm formation. Third, FoxO3a overexpression promoted VSMC phenotypic switching during AAA formation. In addition, we found that FoxO3a overexpression aggravated VSMC autophagy, which has been shown to accelerate VSMC phenotype transitions.

Increasing evidence indicates that FoxO3a is an essential mediator of various vascular diseases^26,32–35^. Despite increasing knowledge of the role of FoxO3a in vascular pathophysiological function, little is known about the role of FoxO3a in AAA. In this study, knockdown of FoxO3a significantly protected against Ang II-induced AAA formation in ApoE^−/−^ mice, inducing significant reductions in the diameter of the suprarenal aorta and elastin degradation scores. These results indicate that FoxO3a may serve as a target of AAA treatment. Studies have reported that Ang II is less effective in inducing the formation of AAA in nonhyperlipidemic mice than in ApoE^−/−^ mice. Therefore, we further determined the role of FoxO3a in the formation and development of AAA by performing gain-of-function experiments in C57BL/6J mice. We found that FoxO3a overexpression substantially hindered the development of Ang II-induced AAA in C57BL/6J mice. Our findings provide evidence that FoxO3a plays an important role in the occurrence and progression of AAA.

Previous studies have shown that VSMC phenotype transitions are observed in the early stages of AAA^18^ and play a central role in the pathogenesis of AAA. FoxO3a has been shown to contribute to abnormal expression of VSMC marker genes^26^. Here, we examined the effect of FoxO3a dysregulation on VSMC phenotypic modulation during Ang-II-induced AAA formation. We found decreased expression of contractile phenotype markers, such as SM22α and α-SMA, and upregulation of synthetic phenotype markers, such as osteopontin (OPN), in FoxO3a-overexpressing mice treated with Ang-II compared to control mice treated with Ang-II. In addition, FoxO3a deficiency was shown to maintain vascular homeostasis during AAA. Our findings indicate that the regulation of VSMC dysfunction by FoxO3a is the key factor in AAA formation. It is well-established that arterial media comprises VSMCs and extracellular matrix and that VSMCs are responsible for the synthesis of extracellular matrix proteins^18,33^. Therefore, VSMC apoptosis is thought to be a key pathological change in the progression of aneurysms^18^. Currently, it is believed that VSMC phenotype transitions are the initiating factor of AAA formation and that VSMC apoptosis is the key driver of the progression of AAA. FoxO3a has been shown to play an important role in inhibiting VSMC proliferation and promoting VSMC apoptosis during atherosclerosis and neointimal hyperplasia^32,33^. Our results and previous studies indicate that FoxO3a might play a central role in the pathogenesis of AAA from onset and throughout disease progression.

VSMCs undergo dedifferentiation from the quiescent, contractile type to the proliferative, synthetic type, which is consistent with the fact that VSMC proliferation is inversely related to VSMC differentiation. Therefore, FoxO3a promotes VSMC phenotypic switching, which indicates that FoxO3a promotes VSMC proliferation. The promotion of VSMC proliferation via elevation of FoxO3a-dependent VSMC phenotypic switching appears to be contradictory to the findings of previous studies demonstrating that VSMC proliferation is inhibited by FoxO3a overexpression during neointimal hyperplasia. A possible reason for this inconsistency is that the suppressive effect of FoxO3a on SMC proliferation and migration via inhibition of proliferative genes is more dominant than its promoting effect on SMC phenotypic switching and that FoxO3a overexpression ultimately inhibits neointimal formation. Genes with similar effects have been reported in previous studies^36,37^. For example, KLF4, a potent repressor of SMC differentiation markers, has been shown to inhibit neointimal formation following vascular injury by reducing VSMC proliferation^36^.

Next, we explored the potential mechanism by which FoxO3a regulates VSMC phenotypic switching. The myocardin (myocd)-dependent transcriptional activation of VSMC contractile genes has been shown to be a major contributor to VSMC homeostasis^21,38,39^. In addition, studies have demonstrated that myocd is a direct target of FoxO3a^22,24^. Consistent with previous in vitro studies, we found that the mRNA expression of myocd was inhibited by FoxO3a overexpression but increased by knockdown of FoxO3a during AAA formation in the present study. A previous study revealed that suppression of autophagy prevents PDGF-induced phenotype switching, indicating that autophagy plays an essential role in VSMC phenotypic switching^40^. FoxO3a has been shown to play a key role in autophagy in various cells^41–43^. Therefore, we assessed whether FoxO3a plays a role in VSMC autophagy during AAA. We found that Ang II-induced VSMC autophagy levels were higher in the FoxO3a overexpression group than the control group. These findings suggest that FoxO3a-induced VSMC autophagy plays a role in VSMC phenotype transitions in the context of AAA.

There are some limitations in this study. First, to better understand the roles of FoxO3a in the formation and development of AAA, the upstream mechanisms by which FoxO3a expression is increased need to be investigated in the future. Second, although we found that FoxO3a overexpression promoted VSMC autophagy induced by Ang II, this evidence is not sufficient to determine the links between VSMC autophagy and VSMC phenotype transitions, which should be demonstrated by in vivo and in vitro experiments with autophagy inhibitors. Third, our study focused on the VSMC phenotypic transitions induced by FoxO3a in AAA; nonetheless, FoxO3a plays a key role in the inhibition of VSMC proliferation and activation of VSMC apoptosis, which remain to be investigated. While FoxO3a may participate in other downstream signaling pathways, we demonstrated that maintaining the VSMC contractile phenotype is essential for the protective effect of FoxO3a deficiency against aortic aneurysm. In addition, we also found that FoxO1 was upregulated in abdominal aortic aneurysm tissues (Supplemental Fig. 6), however, this study only focused on FoxO3, the role of FoxO1 in AAA needs to be further explored in the future.

In summary, we found that knockdown of FoxO3a protects against AAA formation by maintaining VSMC homeostasis. Our findings suggest the potential of FoxO3a as a novel therapeutic target for AAA.

## Supplementary information

Supplementai Figure

Supplementary table
