# Supplementary material for: Loss of FoxO3a prevents aortic aneurysm formation through maintenance of VSMC homeostasis
Source: Cell Death Dis. 2021 Apr 7;12(4):378. doi: 10.1038/s41419-021-03659-y (PMC8027644; doi:10.1038/s41419-021-03659-y)
Supplement: Supplementary file 1 — Supplementai Figure [file 41419_2021_3659_MOESM1_ESM.docx]

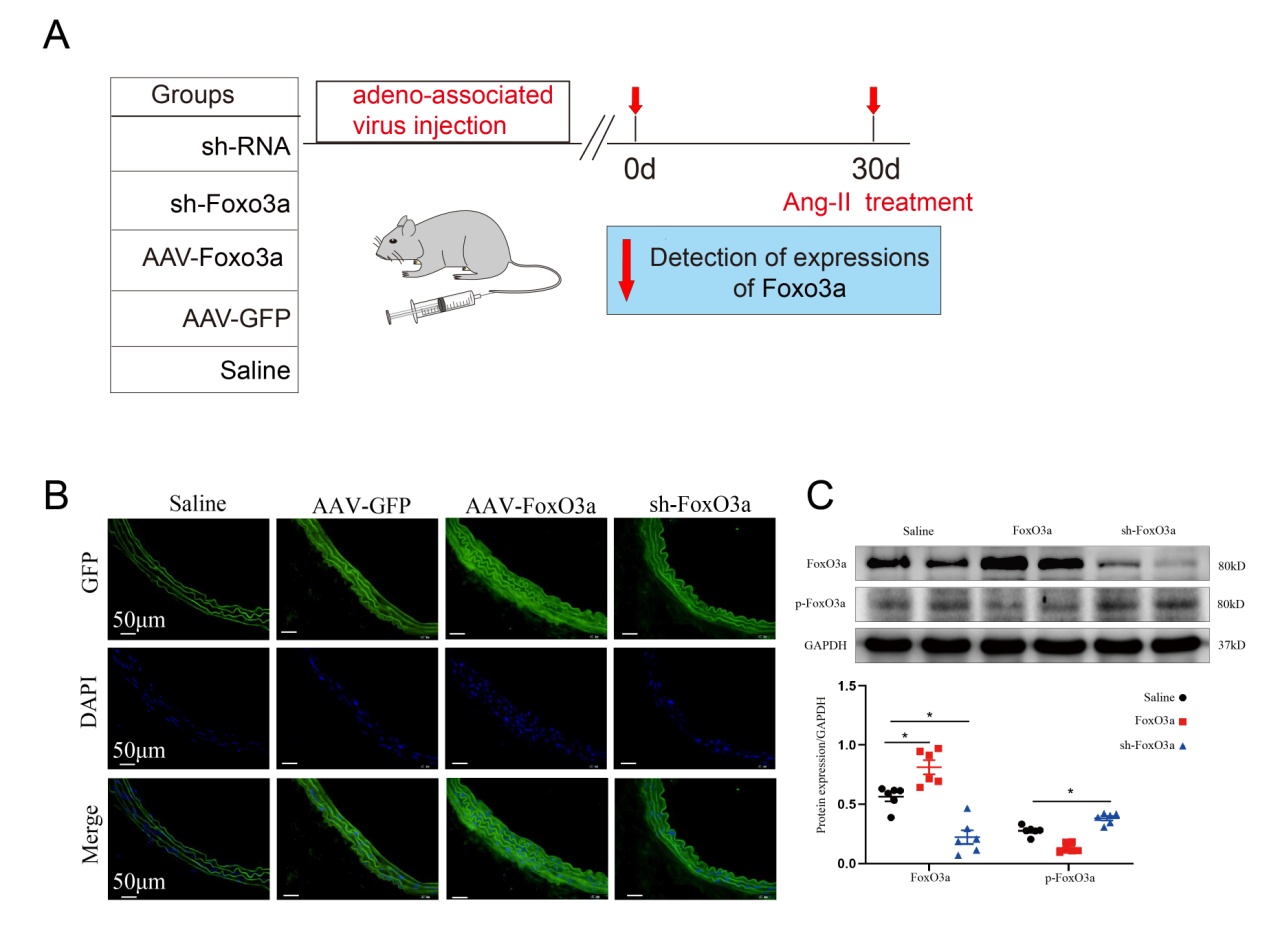


**Supplemental Figure 1. Confirmation of the adeno associated virus transfection efficiency and validation of virus mediating FoxO3a intervention in the suprarenal aortas of mice.**

**A**, Flow diagram of the injection of AAV-mediated FoxO3a knockdown and overexpression. **B**, AAV9 could successfully transfected into VSMCs in vivo. The mice were transfected with AAV- FoxO3a, sh- FoxO3a or saline by the tail vein, respectively. 30 days later, the abdominal aorta of mice was used for frozen section, and then GFP fluorescence was observed under fluorescence fiberscope. (Scale bar=50 μm). **C**, Mice were injected with AAVs and Saline, 30 days after the initial injection, a few mice were sacrificed, and aortic samples were collected to detect aortic FoxO3a expression (n=6). Data are presented as the mean ± SE. ***p*<0.01.

**
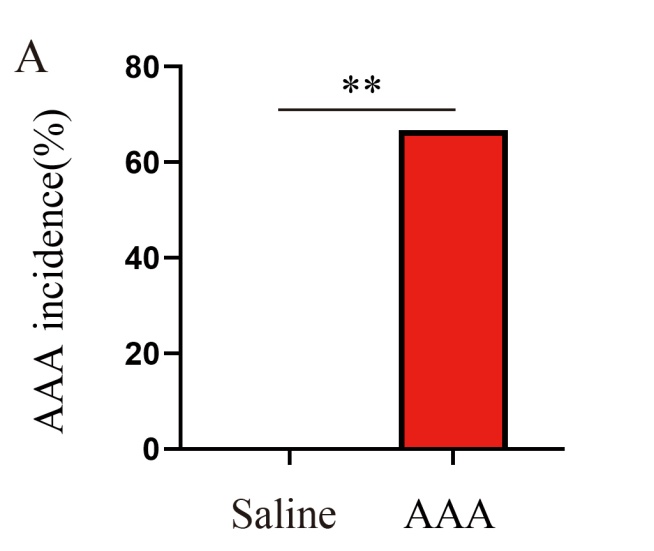
**

**Supplemental Figure 2.** Statistical analysis of the abdominal aortic aneurysm (AAA) incidence in the Ang II-treated ApoE^−/−^ mice. Data are presented as the mean±SE. **p*<0.05, ***p*<0.01.

**
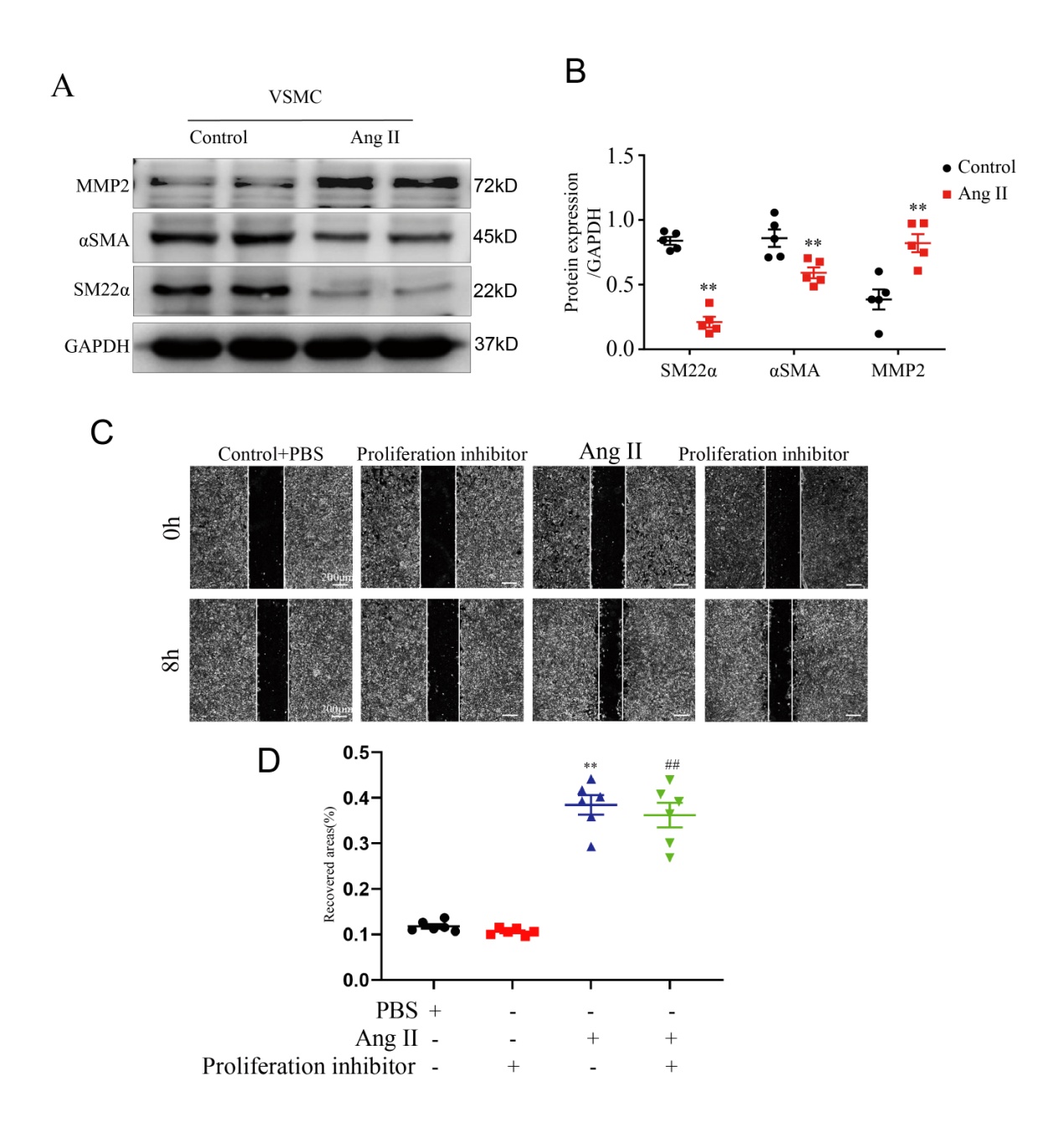
**

**Supplemental Figure 3. A** and **B**, WB and densitometric analysis of the protein levels of α-SMA, MMP2 and SM22α in VSMCs treated with AngII 24h(1μmol/L; n=6). **C and D.** Wound scratch assay was performed to assess VSMC motility and migration within VSMCs after Ang II administration . Data are presented as the mean±SE. **p*<0.05, ***p*<0.01.


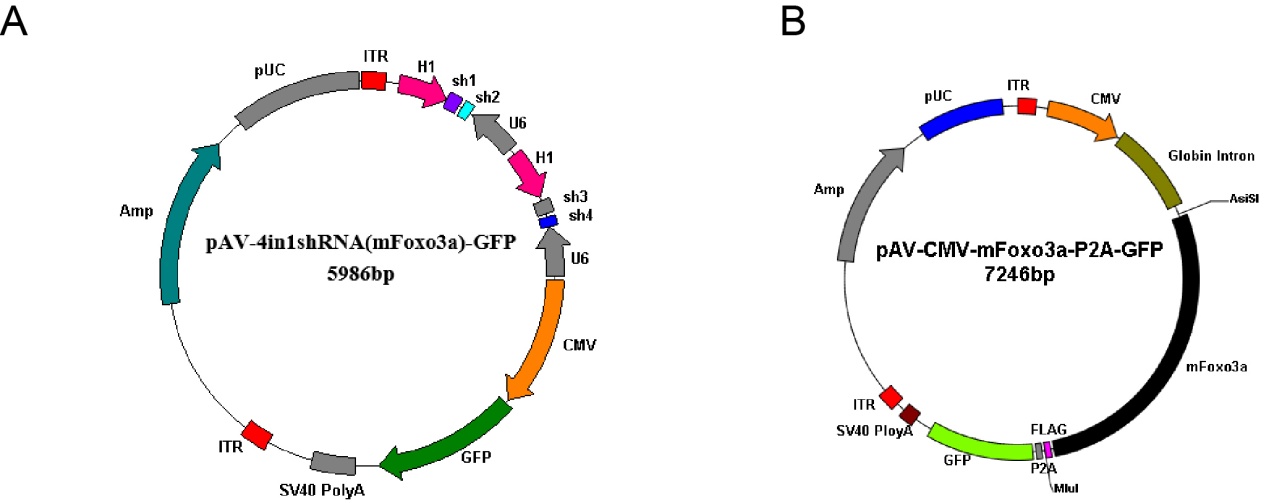


**Supplemental Figure 4.** The full details of the AAV constructs. A, The full details of the AAV-sh-FoxO3a (FoxO3a deficiency); B, The full details of the AAV- FoxO3a (FoxO3a overexpression)


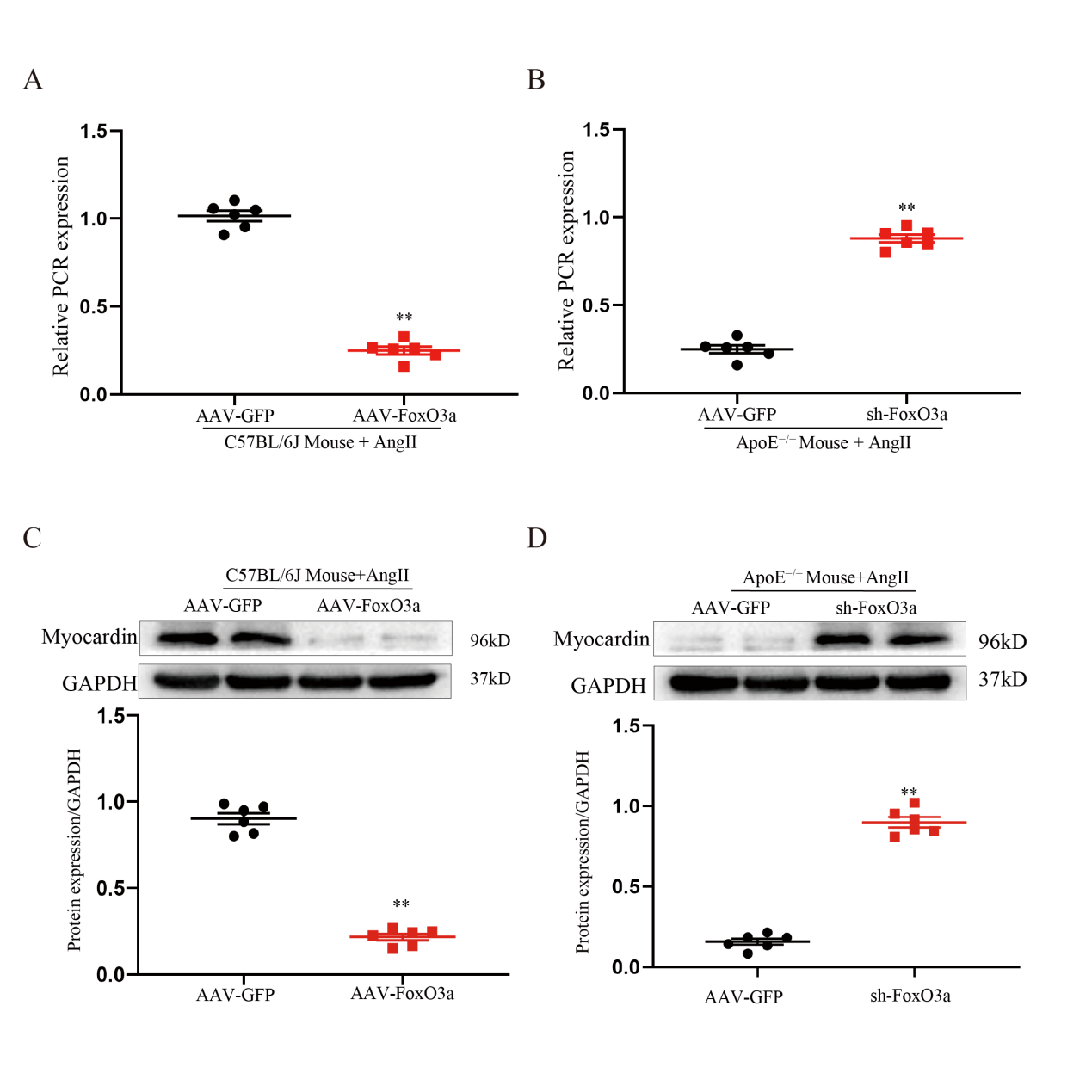


**Supplemental Figure 5. A and B,** qPCR analysis of Myocardin (Myocd) in FoxO3a-overexpressing C57BL/6J mice infused with AngII or FoxO3a-knockdown ApoE^-/-^ mice treated with AngII (n=6). **C and D,** WB and densitometric analysis of the protein levels of Myocardin (Myocd) in FoxO3a-overexpressing C57BL/6J mice infused with AngII or FoxO3a-knockdown ApoE^-/-^ mice treated with AngII (n=6). Data are presented as the mean±SE.**p*<0.05, ***p*<0.01.


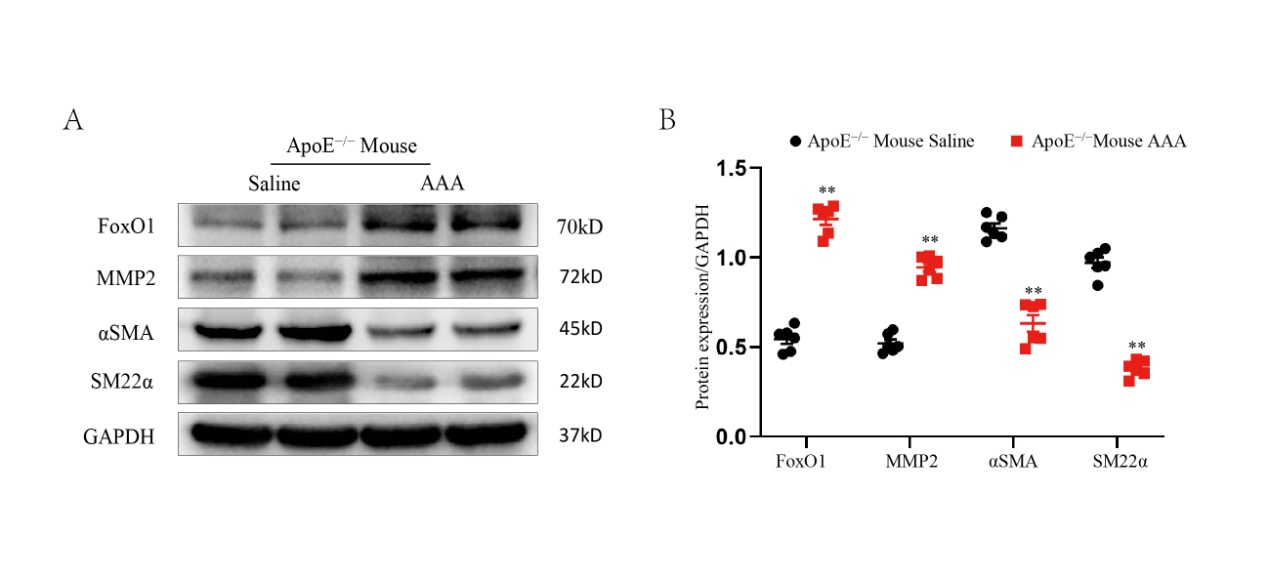


**Supplemental Figure 6.** WB and densitometric analysis of the protein levels of FoxO1, α-SMA, MMP2 and SM22α in ApoE^-/-^ mice infused with AngII (n=6). Data are presented as the mean±SE. **p*<0.05, ***p*<0.01.
