# Supplementary material for: Loss of FoxO3a prevents aortic aneurysm formation through maintenance of VSMC homeostasis
Source: Cell Death Dis. 2021 Apr 7;12(4):378. doi: 10.1038/s41419-021-03659-y (PMC8027644; doi:10.1038/s41419-021-03659-y)
Supplement: Supplementary file 2 — Supplementary table [file 41419_2021_3659_MOESM2_ESM.docx]

**Table I**

**Patient clinical information（n=6）**

| characteristics | AAA |
| --- | --- |
| Ever-smoker | 100% |
| hypertensive | 83.3% |
| hyperlipidemia | 83.3% |
| diabetes | 50% |
| gender | Male(5); Female(1) |
| average age | 59.3±10.4 years |

**Table II**

Antibodies for immunohistochemistry analysis

| name | Vendor or Source | Catalog # |
| --- | --- | --- |
| anti-SM22α | Abcam | ab14106 |
| anti-αSMA | Abcam | ab21027 |
| anti-OPN | Abcam | ab218237 |
| anti- Foxo3a | Abcam | ab154786 |
| anti-IgG | Abcam | ab172730 |

**Table III**

Antibodies for western blots

| name | Vendor or Source | Catalog # |
| --- | --- | --- |
| anti- SM22α | Abcam | ab14106 |
| anti- α-SMA | Abcam | ab32575 |
| anti-OPN | Abcam | ab8448 |
| anti- Foxo3a | Abcam | ab154786 |
| anti- GAPDH | Abcam | ab181602 |
